# The Development of a New Analytical Model for the Identification of Saccharide Binders in Paint Samples

**DOI:** 10.1371/journal.pone.0049383

**Published:** 2012-11-14

**Authors:** Anna Lluveras-Tenorio, Joy Mazurek, Annalaura Restivo, Maria Perla Colombini, Ilaria Bonaduce

**Affiliations:** 1 Dipartimento di Chimica e Chimica Industriale, Università di Pisa, Pisa, Italy; 2 Getty Conservation Institute, Los Angeles, California, United States of America; University of Minho, Portugal

## Abstract

This paper describes a method for reliably identifying saccharide materials in paintings. Since the 3^rd^ millennium B.C., polysaccharide materials such as plant gums, sugar, flour, and honey were used as binding media and sizing agents in paintings, illuminated manuscripts, and polychrome objects. Although it has been reported that plant gums have a stable composition, their identification in paint samples is often doubtful and rarely discussed. Our research was carried out independently at two different laboratories: the Getty Conservation Institute in Los Angeles, USA (GCI) and the Department of Chemistry and Industrial Chemistry of the University of Pisa, Italy (DCCI). It was shown in a previous stage of this research that the two methods give highly comparable data when analysing both reference paint samples and paint layers from art objects, thus the combined data was used to build a large database. In this study, the simultaneous presence of proteinaceous binders and pigments in fresh and artificially aged paint replicas was investigated, and it highlighted how these can affect the sugar profile of arabic, tragacanth, and fruit tree gums. The environmental contamination due to sugars from various plant tissues is also discussed. The results allowed the development of a new model for the reliable identification of saccharide binders in paintings based on the evaluation of markers that are stable to ageing and unaffected by pigments. This new model was applied to the sugar profiles obtained from the analysis of a large number of samples from murals, easel paintings, manuscripts, and polychrome objects from different geographical areas and dating from the 13^th^ century BC to the 20^th^ century AD, thus demonstrating its reliability.

## Introduction

Saccharide materials are known to have been used extensively as binding media for paintings and adhesives for gilding, as testified by several medieval manuscripts from Western Europe and recipe books describing painting techniques and materials [1], [2], [3], [4]. Despite their widespread and documented use, polysaccharide materials have been identified in very few works-of-art [5] such as mural paintings and polychromies in ancient Greece [6], Egypt [7], [8], [9], India [10], and Afghanistan [11]. Saccharide binders have been also identified in the composition of tempera paintings [12], watercolours [13], and between the fibres of a 15^th^ century liturgical vestment [14].

Plant gums (like arabic, tragacanth, and fruit tree) are naturally occurring polysaccharide materials exuded by several species of plants or extracted from the endosperm of some seeds. They are high molecular weight polymers consisting of aldopentoses, aldohexoses, and uronic acids joined together by glycoside bonds [15], [16].

The source of a saccharide binder in a paint sample is usually based on the presence or absence of specific sugars that are identified by the GC-MS analysis of the derivatised sugars after hydrolysis and subsequently compared to reference data [17], [18], [19], [20], [21]. Thermally assisted reactions, silylation [22], [23], hydrolysis, and methylation [24] have all been used successfully in the analysis of reference plant gums and commercial watercolours. Very little is known about the ageing of saccharide materials, although it has been reported that they have a stable composition for up to one hundred years [25]. Quantitative data are rarely reported, but in most cases sugar profiles obtained for paint samples do not quantitatively correspond to any of the profiles obtained from the reference materials [26]. Even though several hypotheses have been suggested, an experimental study has never been carried out. The interactions with the chemicals present in the parchment support [25], the presence of traces of other gums [13] or gum mixtures [18], and monosaccharide interconversion during the ageing process [13], [18] have been suggested as being responsible for the altered chromatographic profiles observed. Biodegradation has also been suggested [18], highlighting similarities with other cases discussed in the literature. In addition, it is well known that amino acids and sugars may react leading to the formation of Maillard reaction products, some of which have been isolated in animal glue [27].

In this paper, the possibility of reliably identifying saccharide materials in paintings is investigated. The research was carried out independently at two laboratories, where two different analytical procedures were used, both based on GC-MS: the Getty Conservation Institute at the Getty Center in Los Angeles, USA (GCI) and the Department of Chemistry and Industrial Chemistry of the University of Pisa, Italy (DCCI). The performances of the two analytical procedures were compared in a previous stage of this research, and it was concluded that the two methods give comparable sugar profiles; whether the samples were simple raw materials, pigmented paint replicas, or paint samples collected from hundreds of centuries old polychrome art objects [28]. In addition, the study produced a common database of sugar profiles that showed how many sources of saccharides can be found in one paint sample [28]. The study also showed that further research was necessary in order to understand the degradation of polysaccharide materials when organic and inorganic materials were present. In order to develop a reliable model for the identification of saccharide binders in paintings, the effect of the simultaneous presence of inorganic and proteinaceous materials and environmental contamination is investigated. In order to evaluate its reliability, this new model is applied to the sugar profiles of more than 50 paint samples from all over the world, spanning more than 30 centuries in age.

## Materials and Methods

### 1. Reagents, raw materials, and reference solutions

Monosaccharides and uronic acids D-(+)-galactose, L-(−)-fucose, L-(+)-arabinose, L-(−)-ramnose, L-(−)-mannose, D-(+)-xylose, D-(+)-glucose, D-glucuronic acid, D-galacturonic acid monohydrate, D-allose, D-glucuronic acid lactone, 2-deoxy-D-ribose, D-psicose, D-tagatose, myo-inositol and mannitol, used as an internal standard, purity 99%, were obtained from Sigma–Aldrich (Milan, Italy).

Trifluoroacetic acid 99% purity, and anhydrous pyridine were from Fluka (Milan, Italy), ethanethiol (ETSH) 99.5%, sodium azide (NaN_3_) 99.5% and *N,O*-bis(trimethylsilyl) trifluoroacetamide (BSTFA) with and without 1% trimethylchlorosilane (TMCS), were from Sigma–Aldrich. Pyridine Sequanal Grade and Trifluoroacetic Acid Sequanal Grade were from Thermo Scientific. The cation/anion exchange resin Zerolit DMF, with the inclusion of an indicator and granulometry comprised between 14 and 52 mesh, was supplied by BDH Chemicals Ltd (UK). Acetic anhydride was from Supelco Inc. Methoxyamine Hydrochloride was from Sigma Aldrich. Ethyl Alcohol, Absolute, 200 Proof was from Spectrum Quality Products, Inc. Water, high purity, Chloroform, Burdick & Jackson, for GC&GC-MS analysis, 4L were from VWR Scientific.

Standard solutions of monosaccharides with concentrations of about 100 ppm were prepared in bidistilled water and 1% sodium azide was added to prevent microbial growth. The solutions were stored at 4°C.

### 2. Reference paint layers

Reference paint layers (pigmented and unpigmented) containing arabic, tragacanth and fruit tree (cherry tree) gums were prepared and subject to natural and artificial ageing in solarbox, weatherometer and thermally. Reference layers of proteinaceous media such as egg, animal glue, milk, and mixtures of proteinaceous and polysaccharides were analysed. The compositions of each paint layer are discussed in detail later in the text.

### 3. Paint samples

The various paint samples that were analysed have different geographical and historical origins, as well as originating from a variety of objects such as easel, mural paintings, and polychromies with different supports. The samples presented here are not chosen in order to discuss the painting technique of the objects they were collected from, rather they are representative of different analytical cases. A detailed sample description is reported in Table 1.

**Table 1:** Description of the paint samples analysed.

Origin	Artwork	Date	samples
Huaca de La Luna, Peru	polychromy on wood	10^th^ century	P-v; P-o; P-c
Near Huaca de La Luna, Peru	mural painting, El Brujo	100 BC-650 AD	P-b m; P-r m; P t m
Constantinople, Turkey	Manuscript	13^th^ century	C u
The Metropolitan Museum of Art, New York, USA	painting “Beggar No. 1”, by Jacob Lawrence, tempera on paper	1938	1938 JL u
National Museum of American Art, Washington, D. C., USA	painting “New Jersey”, by Jacob Lawrence, gouache on paper	1946	1946 JL r
Detroit Institute of Arts, Detroit, MI, USA	painting “Composition for Clarinets and Tin Horn”, by Ben Shahn, tempera on panel	1951	1951 BS
Cave 85, Mogao China	mural painting	6^th^ century	MS-8-D; MS-4-D; M15 -5; MS-2-U; M-o.red
Nefertari Tomb, Luxor, Egypt	mural painting	13^th^ century BC	Nef-y; Nef-r; Nef-b
Egypt	Cartonnage	about 100 AD	UC4598E-A
Egypt	Cartonnage	512-351 BC	USC9429-g; USC9429-c; USC9429-y
Egypt	polychromy on wooden male head (Skirball	about 100 AD	A939
Egypt	polychromy on wood ushabti	Unknown	USC9402
Temple of the Winged Lions, Petra, Jordan	polychromy on pottery	1^st^ century	P-b
Tomb 826, Petra, Jordan	mural painting	1^st^ century	P-CO82654B
Chiesa della Missione, Mondovi, Italy	mural painting by A. Pozzo	17^th^ century	M-op
Vardala Palace, Malta	painted architecture	1910	VP75
Tomb of Saint Anthony, Basilica of Saint Anthony from Padua, Padua, Italy	dark painted decoration on marble high-relief “St. Anthony receiving the Franciscan habit”, by Antonio Minello	1517	Pdv 3
Tomb of Saint Anthony, Basilica of Saint Anthony from Padua, Padua, Italy	dark painted decoration on marble high-relief “The young man resurrected by the Saint”, by Danese Cattaneo, completed by Girolamo Campagna	1573	Pdv 4
Tomb of Saint Anthony, Basilica of Saint Anthony from Padua, Padua, Italy	dark painted decoration on marble high-relief “The miracle of the reattached foot”, by Tullio Lombardo	1504	Pdv 6
Monumental Cemetery, Pisa, Italy	mural painting, superficial organic patina	20^th^ century	patina superficial
Byzantine Christian Museum of Greece, Athens, Greece	panel painting “Panagia Kardiotissa”, Aggelos	15^th^ century	pk2; pk4; pk8; pk9; pkB2
Eastern Buddha, Bamiyan Valley, Afghanistan	restoration patina on polychrome clay sculpture	6^th^ century	214-int
	polychromy on clay sculpture		214-6-5; 214-4-3
Western Buddha, Bamiyan Valley, Afghanistan	polychromy on clay sculpture, fragment	6^th^ century	97-2; 168-2; 14-7-5-4; 108-4; 108-3
Buddhist temple of Shuilu'an (Shaanxi Province, China)	polychromy on clay sculpture	16^th^ century	SL-R10 g; SL-R10 r; SL-R10 w; SL-R10 c; SLGR02; R08B

### 4. Apparatus

A microwave oven MLS MEGA Milestone 1200 W (Milestone Microwave Laboratory System, Monroe, CT, USA) was used at DCCI. Hydrolysis conditions were: power 500 W, temperature 120°C, duration 20 min.

6890N GC System Gas Chromatograph (Agilent Technologies, Palo Alto, CA, USA), coupled with a 5975 Mass Selective Detector (Agilent Technologies, Palo Alto, CA, USA) single quadrupole mass spectrometer, equipped with a PTV injector were used at DCCI. The instrumental parameters are described elsewhere [18], [29]

ARC10-22 speed vacuum system with a refrigerated trap RCT 90 from Thermo Electron Corporation (St. Herblain, France) was used at DCCI to dry the samples.

Reacti-Vap Evaporator, Reacti-Block A-1, Racti-Therm Heating Module and Reacti-Therm Thermometer, from Thermo Scientific were used at GCI.

A Solarbox 1500e RH (Erichsen, Milano, Italy), purchased from Erichsen (Germany), was used for the artificial ageing of the reference paint layers at DCCI. The ageing conditions were as follows: 20°C, relative humidity 50%, excitation with a Xenon lamp, wavelength 280–400 nm, power 400 W, 30 days. A Soda-lime glass UV filter was used to simulate indoor exposure. Irradiation uniformity was guaranteed by a parabolic reflector chamber with the Xenon Lamp in the focus.

A Weatherometer Ci400 with soda lime filters, irradiance of 0.5 w/m^2^, relative humidity of 50%, 40°C chamber temperature, and black panel temperature of 55°C was used at GCI. Exposure time was six weeks. Dimensions of the exposure chamber were: height 198 cm, width 127 cm, depth 102 cm.

Artificial ageing in the oven was performed for 500 hours at 80°C at GCI.

### 5. Analytical procedures

The GCI procedure is based on the methoxylamine acetate derivatisation of neutral sugars (aldoses and ketoses) obtained from saccharide materials after hydrolysis. The DCCI procedure is based on the analysis of the mercaptal derivatives of the parent aldoses and uronic acids obtained after microwave assisted hydrolysis. The details of both procedures are published elsewhere [28].

## Results and Discussion

### 1. Effect of the simultaneous presence of inorganic materials

In a previous stage of this research, it was suggested that the presence of a pigment in a paint layer containing a polysaccharide gum might cause modifications upon ageing [28]. For this reason, the effect of ageing in the presence of some pigments was investigated. The relative sugar composition of each paint replica analysed is presented in Table 2, Table 3 and Table 4 in relation to the gum, pigment present, and ageing process.

**Table 2:** Relative sugar composition of arabic gum paint replicas analysed, in relation to the pigment present, and ageing (the results are the averages of three replicates).

Pigment	ageing	xylose	arabinose	rhamnose	fucose	galacturonic acid	glucuronic acid	mannose	galactose	method	correlation coefficient	decisional scheme
-	n	0.2	37.2	18.3	0.1	-	-	0.0	44.3	GCI	1.00	A
	n	0.0	25.7	13.3	0.0	0.0	11.7	0.0	49.3	DCCI	1.00	A
	W	0.2	37.8	18.7	0.1	-	-	0.0	43.1	GCI	1.00	A
	O	0.6	39.9	19.4	0.0	-	-	0.0	40.2	GCI	0.99	A
	SB 2	0.0	36.0	17.7	0.0	0.0	11.2	0.0	48.7	DCCI	0.99	A
Hg (vermilion)	n	0.4	38.8	18.5	0.1	-	-	0.0	42.2	GCI	1.00	A
	W	0.6	39.9	19.3	0.0	-	-	0.0	40.2	GCI	0.99	A
	O	0.6	38.8	17.9	0.2	-	-	0.0	42.6	GCI	1.00	A
Si (ultramarine)	n	0.3	42.1	23.8	0.0	-	-	0.0	33.8	GCI	0.95	A
	W	0.6	43.7	21.6	0.0	-	-	0.0	34.2	GCI	0.95	A
	O	0.3	42.0	21.3	0.3	-	-	0.0	36.2	GCI	0.97	A
	n	0.3	31.3	16.5	0.0	0.0	9.7	0.2	42.0	DCCI	0.98	A
	SB 1	0.3	30.6	14.1	0.0	0.0	3.6	0.2	51.2	DCCI	0.98	A
	SB 2	0.4	28.4	12.5	0.0	0.0	7.4	0.2	51.1	DCCI	0.99	A
Cu (malachite)	n	0.2	37.0	19.4	0.0	-	-	0.0	43.5	GCI	1.00	A
	W	0.6	36.3	19.0	0.0	-	-	0.0	44.1	GCI	1.00	A
	O	0.0	40.4	20.2	0.0	-	-	0.0	39.4	GCI	0.99	A
Cu (copper acetate)	n	0.3	39.4	11.8	0.0	0.0	4.8	0.1	43.5	DCCI	0.94	A
	SB 1	0.5	31.9	11.5	0.0	0.0	7.2	0.2	48.7	DCCI	0.99	A
	SB 2	0.1	31.9	8.9	0.0	0.1	6.2	0.1	52.7	DCCI	0.98	A
C (vine black)	n	0.1	36.3	16.4	0.1	-	-	0.1	46.9	GCI	1.00	A
	W	0.0	36.6	16.1	0.0	-	-	0.2	47.0	GCI	1.00	A
	O	0.0	36.1	16.6	0.1	-	-	0.1	47.2	GCI	1.00	A
Pb (lead white)	n	0.7	54.9	26.9	0.2	-	-	0.0	17.3	GCI	0.73	A
	W	0.3	46.2	22.8	0.2	-	-	0.0	30.5	GCI	0.92	A
	O	0.3	47.3	23.1	0.0	-	-	0.0	29.3	GCI	0.90	A
Pb (minium)	n	0.4	33.7	15.5	0.0	0.0	11.8	0.2	38.3	DCCI	0.96	A
	SB 1	0.1	25.3	14.2	0.0	0.0	7.8	0.1	52.5	DCCI	1.00	A
	SB 2	0.1	26.2	10.9	0.0	0.0	11.9	0.1	50.8	DCCI	1.00	A
Fe (yellow ochre)	n	0.1	36.0	16.5	0.1	-	-	0.1	47.2	GCI	1.00	A
	W	0.1	36.0	16.9	0.1	-	-	0.2	46.8	GCI	1.00	A
	O	0.0	35.4	16.2	0.1	-	-	0.1	48.1	GCI	1.00	A
Fe (red bole)	n	0.2	23.0	11.9	0.0	0.0	8.7	0.4	55.8	DCCI	0.99	A
	SB 1	0.0	29.6	16.9	0.0	0.0	3.5	0.0	50.0	DCCI	0.98	A
	SB 2	0.1	26.7	12.2	0.0	0.1	11.4	0.1	49.4	DCCI	1.00	A

*Arabic (A), tragacanth (T), fruit tree (cherry tree) (F, natural ageing (n), artificial ageing in solarbox (sb), weatherometer (w), and thermal (t). natural, at GCI (2 years) – n; natural, at DCCI (2 years) – n; artificial in the oven for 6 weeks – O; artificial in the Weatherometer for 6 weeks – W; artificial in the Solar box for 1 week – SB 1; artificial in the Solar box for 4 weeks – SB 2.*

**Table 3:** Relative sugar composition of tragacanth gum paint replicas analysed, in relation to the pigment present, and ageing (the results are the averages of three replicates).

Pigment	ageing	xylose	arabinose	rhamnose	fucose	galacturonic acid	glucuronic acid	mannose	galactose	method	correlation coefficient	decisional scheme
-	n	29.2	47.7	1.5	11.1	-	-	0.0	10.5	GCI	1.00	T
	n	23.5	40.2	2.3	8.0	14.1	0.6	0.0	11.3	DCCI	1.00	T
	W	29.3	47.1	1.6	11.1	-	-	0.0	10.9	GCI	1.00	T
	O	29.7	46.9	1.6	10.9	-	-	0.0	11.0	GCI	1.00	T
	SB 2	22.5	39.0	3.4	8.3	15.1	0.4	0.0	11.1	DCCI	1.00	no
C (vine black)	n	28.5	47.4	1.7	11.0	-	-	0.0	11.3	GCI	1.00	T
	W	27.8	44.9	4.6	10.8	-	-	0.0	12.0	GCI	1.00	T
	O	28.6	45.5	3.6	10.7	-	-	0.0	11.7	GCI	1.00	T
Pb (lead white)	n	24.7	54.7	1.2	16.1	-	-	0.0	3.3	GCI	0.97	T
	W	25.4	53.2	1.1	14.7	-	-	0.0	5.6	GCI	0.98	T
	O	29.7	49.4	1.7	12.5	-	-	0.0	6.7	GCI	1.00	T
Pb (minium)	n	33.0	39.1	2.9	7.4	1.4	0.6	0.0	15.6	DCCI	0.92	T
	SB 1	30.9	41.3	3.4	7.0	0.9	0.3	0.0	16.2	DCCI	0.92	no
	SB 2	16.2	59.5	3.1	5.3	0.2	0.1	0.0	15.6	DCCI	0.91	no
Hg (vermilion)	n	27.6	49.6	1.6	10.5	-	-	0.0	10.6	GCI	1.00	T
	W	25.9	51.7	2.0	10.0	-	-	0.0	10.4	GCI	0.99	T
	O	28.5	47.7	2.7	8.8	-	-	0.0	12.3	GCI	1.00	T
Si (ultramarine)	n	20.3	57.1	1.2	16.6	-	-	0.0	4.8	GCI	0.95	T
	W	18.7	59.1	0.8	14.6	-	-	0.0	6.9	GCI	0.95	no
	O	24.1	54.8	1.2	13.9	-	-	0.0	6.0	GCI	0.98	T
	n	25.2	45.8	3.0	9.5	0.2	0.3	0.0	16.0	DCCI	0.93	no
	SB 1	20.1	46.2	2.6	4.1	0.0	0.0	0.0	27.0	DCCI	0.87	no
	SB 2	25.6	47.5	3.0	8.4	0.0	0.2	0.0	15.2	DCCI	0.94	no
Fe (yellow ochre)	n	27.3	50.8	1.3	12.5	-	-	0.0	8.1	GCI	0.99	T
	W	27.7	46.8	3.9	11.0	-	-	0.0	10.5	GCI	1.00	T
	O	27.8	44.7	3.6	10.8	-	-	0.0	13.1	GCI	1.00	T
Fe (red bole)	n	30.5	37.9	2.6	12.3	4.1	0.4	0.0	12.2	DCCI	0.94	no
	SB 1	26.5	39.4	3.5	10.4	5.1	0.0	0.0	14.9	DCCI	0.96	no
	SB 2	29.1	43.4	2.9	11.3	0.1	0.2	0.3	12.6	DCCI	0.93	no
Cu (malachite)	n	7.2	72.0	0.9	18.6	-	-	0.0	1.3	GCI	0.85	no
	W	3.3	71.2	1.4	23.7	-	-	0.0	0.5	GCI	0.80	T
	O	10.1	67.9	1.8	20.2	-	-	0.0	0.0	GCI	0.86	T
Cu (copper acetate)	n	13.2	37.8	5.6	3.2	4.6	9.2	0.0	26.5	DCCI	0.78	T
	SB 1	9.7	41.5	6.8	3.5	3.1	4.7	0.0	30.8	DCCI	0.75	T
	SB 2	10.9	47.6	4.1	2.1	0.4	3.1	0.0	31.8	DCCI	0.77	no

*Arabic (A), tragacanth (T), fruit tree (cherry tree) (F, natural ageing (n), artificial ageing in solarbox (sb), weatherometer (w), and thermal (t). natural, at GCI (2 years) – n; natural, at DCCI (2 years) – n; artificial in the oven for 6 weeks – O; artificial in the Weatherometer for 6 weeks – W; artificial in the Solar box for 1 week – SB 1; artificial in the Solar box for 4 weeks – SB 2.*

**Table 4:** Relative sugar composition of fruit gum paint replicas analysed, in relation to the pigment present, and ageing (the results are the averages of three replicates).

Pigment	ageing	xylose	arabinose	rhamnose	fucose	galacturonic acid	glucuronic acid	mannose	galactose	method	correlation coefficient	decisional scheme
-	n	11.8	54.3	1.3	0.1	-	-	2.2	30.2	GCI	1.00	F
	n	7.0	48.7	2.1	0.0	0.0	5.9	1.8	34.4	DCCI	1.00	F
	W	12.4	54.9	1.4	0.1	-	-	1.2	30.1	GCI	1.00	F
	O	10.5	48.4	1.2	0.1	-	-	3.1	36.6	GCI	0.99	F
	SB 2	6.1	43.2	2.1	0.0	0.0	9.0	2.7	36.9	DCCI	0.99	F
Pb (lead white)	n	17.0	71.9	2.1	0.0	-	-	1.5	7.5	GCI	0.87	F
	W	18.7	67.0	2.2	0.0	-	-	3.3	8.8	GCI	0.87	F
	O	14.7	55.9	0.0	0.0	-	-	2.9	26.5	GCI	0.99	no
Pb (minium)	n	6.8	53.0	1.1	0.1	0.0	3.7	3.8	31.5	DCCI	0.99	F
	SB 1	5.1	50.7	0.9	0.1	0.0	11.4	5.2	26.6	DCCI	0.98	no
	SB 2	3.6	53.9	1.0	0.0	0.0	3.6	3.5	34.3	DCCI	0.99	F
Si (ultramarine)	n	8.6	54.2	2.5	0.0	-	-	1.4	33.3	GCI	1.00	F
	W	14.9	79.3	1.8	0.5	-	-	1.8	1.8	GCI	0.83	F
	O	10.1	53.6	1.4	0.0	-	-	2.9	31.9	GCI	1.00	F
	n	6.0	57.7	1.2	0.0	0.0	6.6	2.8	25.7	DCCI	0.97	F
	SB 1	7.8	55.6	1.2	0.0	0.0	7.2	2.6	25.6	DCCI	0.98	F
	SB 2	5.5	57.9	1.0	0.0	0.0	3.6	3.1	28.9	DCCI	0.98	F
C (vine black)	n	12.0	51.0	1.7	0.0	-	-	1.3	34.0	GCI	1.00	F
	W	16.4	54.1	1.6	0.0	-	-	1.6	26.2	GCI	0.99	F
	O	9.8	48.4	1.3	0.0	-	-	3.9	36.6	GCI	0.99	F
Hg (vermilion)	n	10.4	45.8	1.0	0.0	-	-	2.3	40.4	GCI	0.97	F
	W	10.4	40.4	1.1	0.0	-	-	3.2	45.0	GCI	0.92	F
	O	10.2	32.2	0.0	0.0	-	-	3.4	54.2	GCI	0.80	no
Fe (yellow ochre)	n	10.6	49.8	2.2	0.0	-	-	3.1	34.4	GCI	1.00	F
	W	12.2	53.1	2.0	0.0	-	-	2.0	30.6	GCI	1.00	F
	O	12.0	45.8	1.2	0.0	-	-	3.6	37.3	GCI	0.98	F
Fe (red bole)	n	3.7	51.1	0.9	0.0	0.1	12.9	3.2	28.2	DCCI	0.98	no
	SB 1	6.8	58.7	1.4	0.0	0.0	5.2	2.1	25.9	DCCI	0.97	F
	SB 2	5.0	57.7	1.0	0.0	0.0	4.1	3.0	29.3	DCCI	0.98	F
Cu (malachite)	n	8.3	83.0	1.8	0.3	-	-	0.0	6.7	GCI	0.87	no
	W	12.5	79.2	4.2	0.0	-	-	0.0	4.2	GCI	0.85	no
	O	9.1	90.9	0.0	0.0	-	-	0.0	0.0	GCI	0.83	no
Cu (copper acetate)	n	3.8	60.8	0.7	0.1	0.0	7.4	3.4	23.8	DCCI	0.96	no
	SB 1	6.6	52.8	0.6	0.0	0.0	12.1	3.5	24.4	DCCI	0.97	no
	SB 2	2.7	61.3	0.5	0.0	0.0	1.7	2.6	31.2	DCCI	0.98	no

*Arabic (A), tragacanth (T), fruit tree (cherry tree) (F, natural ageing (n), artificial ageing in solarbox (sb), weatherometer (w), and thermal (t). natural, at GCI (2 years) – n; natural, at DCCI (2 years) – n; artificial in the oven for 6 weeks – O; artificial in the Weatherometer for 6 weeks – W; artificial in the Solar box for 1 week – SB 1; artificial in the Solar box for 4 weeks – SB 2.*

An analysis of the paint layers containing plant gums without pigments showed that the molecular profile of each gum was maintained after artificial ageing. If modifications of the macromolecular structure of the polysaccharide fraction occurred, either hydrolysis and a subsequent molecular weight decrease or cross-linking with a subsequent molecular weight increase, the recovery of sugars after the hydrolysis step was not significantly altered. For example, the recovery of the three gums (based on the sum of the absolute amounts of sugar detected in the chromatograms with respect to the sample weight) from unaged paint layers, using the DCCI procedure, is 65% for Arabic gum, 23% for tragacanth gum and 62% for fruit tree gum. For aged paint layers, the recoveries are 92% for Arabic gum, 23% for tragacanth gum and 62% for fruit tree gum.

Alternatively, the composition of paint layers containing pigments after artificial ageing showed that both qualitative and quantitative changes occur in the sugar composition due to ageing conditions and the pigment present. Quantitatively and qualitatively speaking, some sugars appear stable under ageing while others do not.

As a general conclusion, it can be stated that arabic gum is quite stable, showing a constant qualitative sugar profile. Tragacanth and fruit tree gums are the most subjected to modifications in the sugar profile. Peaks ascribable to sugar degradation products were not detected in the chromatograms.

Calculating the correlation coefficient revealed that the paint replicas did not always give good correlation coefficients: 53 out of 105 samples showed an excellent correlation coefficient (0.99–1.00), 21 a good correlation coefficient (0.96–0.98), and 31 a poor correlation coefficient (<0.96). Poor correlation coefficients were generally obtained for the three gums when Si, Cu and Pb were present. Fe also had an influence on tragacanth gum, while fruit tree was affected by Hg. These results show that using the correlation coefficient as the only means to identify the source of plant gum is not reliable, even when a very simple system is under investigation such as mixture of one plant gum and one pigment. The observed effects of these pigments on the saccharide profiles of plant gums is probably due to both a pigment interaction during the derivatisation procedure, as well as degradation from artificial aging conditions.

The changes observed in the relative sugar profiles can be summarized as follows:

arabinose was present and relatively abundant in all samples, although subject to changes in the relative amounts depending on the pigment and ageing (ranging from 28.4% to 90.9%);
galactose was present in all samples, although subject to changes in the relative amounts depending on the pigment and the ageing (ranging from 1.3% to 55.8%). The only exceptions were the fruit tree and tragacanth gum samples, which contained malachite and were artificially aged in the oven and weatherometer;
xylose, in arabic gum, was present in small amounts with a relative content always below 1.0%; it was present and relatively abundant in all samples in tragacanth (ranging from 3.3% to 30.0%) and fruit tree (ranging from 3.7% to 18.7%) gums, although subject to changes in the relative amounts depending on the pigment and ageing;
fucose, in arabic and fruit tree gums, showed very low relative content, which was always below 1.0%; it was relatively abundant in all samples in tragacanth gum, although subject to changes in the relative amounts depending on the pigment and ageing (ranging from 2.1% to 28.7%);
mannose, in arabic and tragacanth gums, showed a very low relative content and was always below 1.0%; in fruit tree gum it was present above 1.0% in all samples, although subject to changes in the relative amounts depending on the pigment and ageing (ranging from 1.2% to 5.2%), with the exception of all the samples containing malachite (0.0%);
rhamnose was relatively abundant in the arabic gum used as reference material, and although subject to changes in the relative amounts depending on the pigment and ageing, it was present and relatively abundant in all samples (ranging from 8.9% to 26.9%); in tragacanth and fruit three gums rhamnose showed quite a low relative content, always below 6.8%, and depending on the pigment and ageing conditions it also often dropped below 1.0%;
galacturonic acid in arabic and fruit tree gums was present in small amounts, with a relative content always below 1.0%; in tragacanth gum it was subject to considerable changes, ranging from 0.0% to 15.1%, depending on the pigment and ageing conditions;
glucuronic acid in arabic and fruit tree gum was present and relatively abundant in all samples, although subject to changes in the relative amounts depending on the pigment and ageing (ranging from 3.6% to 12.9%); in tragacanth gum its relative content stayed below 1.0%, with the exception of all the samples containing copper acetate, showing an increase to values much higher than 1.0% (ranging from 0.0% to 15.1%).

The qualitative changes in the sugar profile of the samples analysed highlight the limitations in identifying the source of the plant gum with the decisional scheme previously reported in the literature [18] in 22 cases (12 tragacanth gum samples and 10 fruit tree samples), and shows the unreliability of the decisional scheme adopted in identifying the source of plant gum. On the other hand, based on the changes observed in the sugar profiles discussed above, it is possible to draw some conclusions that can be used to build a new decisional scheme. Arabinose and galactose are present in all three of the plant gums analysed. However, rhamnose and the uronic acids must be excluded from the decisional scheme due to their instability under ageing in the presence of some pigments, even though their presence can help with interpreting the data. The distinction between arabic and tragacanth/fruit tree gums can thus be based on xylose: a content lower than 1.0% suggests that arabic gum is present, while a relative content higher than 1.0% suggests that tragacanth or fruit tree gum are present. Fucose and mannose help us to distinguish between tragacanth and fruit tree gums: a fucose content higher than 1.0% and a mannose content lower than 1.0% indicates that tragacanth is present, while a fucose content lower than 1.0% and a mannose content higher than 1.0% points to the occurrence of fruit tree gum.

Finally, it is important to highlight that the relative content of xylose has never been found to be higher than arabinose. This strongly suggests that external contamination must be considered as a source of saccharide material when the profile reported in the literature has a xylose content higher than that of arabinose [18].

### 2. Effect of the simultaneous presence of other organic materials

#### 2.1 Proteinaceous materials

Results from a previous stage of this research showed that sugars are found in many materials that can be encountered in a paint sample, such as organic colorants, plant resins and proteinaceous binders [28]. This indicates that a mixture of binders, which is extremely common in paintings, may lead to chromatogram profiles that are difficult to interpret when the overall composition of the organic materials is unknown. Given the widespread use of proteinaceous materials as paint binders [1], we investigated the sugar profiles that can be obtained when proteinaceous media and plant gums are simultaneously present. Reference paint layers of polysaccharide gums and proteinaceous materials (mixtures approximately 1∶1) were analysed after two years of natural ageing. Data are presented in Table 5, compared to the theoretical (th) values obtained from calculating the sugar profile of a 1∶1 mixture of the saccharide and proteinaceous binders. The sugar profile and saccharide content of the gums and proteinaceous materials have been published elsewhere [28]

**Table 5:** Relative percentage sugar content of paint layers containing a mixture of plant gums and proteinaceous materials: a comparison of theoretical sugar profiles with experimental ones.

gum	proteinaceous material	sugar profile	xylose	arabinose	rhamnose	fucose	galacturonic acid	glucuronic acid	glucose	mannose	galactose
**arabic**	**egg**	*theoretical*	0.0	23.5	11.4	0.0	0.0	10.6	1.8	7.2	45.5
		experimental	0.0	31.3	14.8	0.0	0.0	10.6	0.5	2.0	40.8
	**animal glue**	*theoretical*	0.0	25.9	12.5	0.0	0.0	11.6	0.6	0.0	49.4
		experimental	0.0	35.6	15.2	0.0	0.0	11.6	0.1	0.2	35.2
	**milk**	*theoretical*	0.0	22.4	10.8	0.0	0.0	10.1	5.7	0.5	50.6
		experimental	0.0	30.5	14.3	0.0	0.0	10.8	8.7	0.0	35.7
**tragacanth**	**egg**	*theoretical*	16.5	28.5	1.9	6.0	12.0	0.7	12.4	10.5	11.6
		experimental	20.7	33.1	1.1	4.0	2.0	0.6	12.1	16.8	9.8
	**animal glue**	*theoretical*	18.9	32.6	2.1	6.9	13.7	0.9	12.0	0.0	12.9
		experimental	22.0	37.4	1.8	6.3	3.0	0.5	12.6	0.1	16.3
	**milk**	*theoretical*	15.3	26.4	1.7	5.6	11.1	0.7	17.0	0.7	21.5
		experimental	3.8	6.2	0.2	1.1	1.4	0.5	46.2	0.4	40.2
**fruit tree**	**egg**	*theoretical*	6.5	37.9	1.8	0.0	0.0	4.4	2.1	10.4	37.0
		experimental	15.5	45.3	1.2	0.0	0.0	5.2	1.4	6.5	25.1
	**animal glue**	*theoretical*	7.2	42.1	2.0	0.0	0.0	4.9	0.7	2.3	40.8
		experimental	14.7	45.2	1.7	0.0	0.0	5.7	1.4	2.3	29.2
	**milk**	*theoretical*	6.1	35.8	1.7	0.0	0.0	4.2	6.4	2.2	43.6
		experimental	5.8	21.0	0.5	0.0	0.2	2.7	21.7	1.3	46.9

The experimental results show quite a good agreement with the theoretical profiles calculated, although some quantitative differences can be observed. These can mainly be ascribed to the heterogeneity of the prepared paint layers and the fact that the experimental mixtures were only approximately 1∶1. Although browning reactions [30] were observed after hydrolysis, this does not seem to have significantly influenced the qualitative molecular patterns.

The data clearly show that the sugar profile of a plant gum may appear to be modified when a proteinaceous binder is present. It is important to note that although egg has a relatively low content of saccharide material (about 1%); it is clearly not negligible when it is mixed in similar proportions to saccharide materials. Therefore, mannose cannot be considered as a marker for fruit tree gum as it might clearly derive from the simultaneous presence of egg. This indicates that the presence of egg must be known and taken into consideration when mannose is present.

#### 2.2. Wood, paper, straw, and other materials originating from plant tissues

Wood is a material that commonly contaminates a polychrome, as it often is the support of the object (such as a wooden painted object or a manuscript on paper), or it might be present in the paint or preparation layers (such as straw, which was often mixed with the paint to add cohesion to the preparatory layers). If contamination from the wooden material is present in the painting, the contribution of its sugar composition [28], [31], [32], [33] will affect the sugar profile of the polysaccharide gum present. Moreover, past research highlighted how polysaccharides from plant tissues are present in deposition of rain and snow, representing more than 50% of its total organic carbon [34]. Although this needs further investigation, it can be reasonably expected that polysaccharides from plant tissues (wood, straw, paper, etc…) may be present in the particulate matter in all environments, both indoor and outdoor. In order to make an estimate of what could be the effect of a contamination from plant tissues, the sugar composition of arabic, tragacanth, and fruit tree gums contaminated by 10% of the saccharide content of softwood and hardwood was calculated and the sugar profiles are reported in Table 6 (the sugar profile and saccharide content of the gums and wood have been published elsewhere [28]).

**Table 6:** Calculated sugar profile of arabic, tragacanth and fruit tree gums contaminated by the 10% of the saccharide content of softwood and hardwood.

gum	wood	xylose	arabinose	rhamnose	fucose	galacturonic acid	glucuronic acid	glucose	mannose	galactose
**arabic**	**softwood**	7.5	26.2	13.1	0.0	0.0	12.0	1.6	0.1	49.5
	**hardwood**	2.4	26.7	13.0	0.0	0.1	12.0	2.7	3.2	49.9
**fruit tree**	**softwood**	12.5	35.2	2.1	0.0	0.0	4.0	1.6	2.1	52.5
	**hardwood**	7.4	35.7	2.0	0.0	0.1	4.0	2.7	5.2	52.9
**tragacanth**	**softwood**	26.5	31.2	2.1	6.0	17.0	1.0	13.6	0.1	12.5
	**hardwood**	21.4	31.7	2.0	6.0	17.1	1.0	14.7	3.2	12.9

The table clearly shows that xylose cannot be used to distinguish between arabic and fruit tree or tragacanth gums, and mannose cannot be used to identify fruit tree gum when softwood is present. Moreover, when the contamination levels are higher than 10%, then a much higher content of xylose must be expected and this might explain those cases reported in the literature where the ratio xylose/arabinose is found to be higher than one [18].

### 3. A new model to identify the polysaccharide materials present

On the basis of the results of the effects of pigments, the simultaneous presence of proteinaceous media, and the contamination from wood and other plant tissues, it is possible to develop four new decisional schemes to identify the source of a plant gum that can be used in four different cases:

- there are no proteinaceous materials simultaneously present and there seems to be no contamination. As shown in the previous paragraph, it is not always possible to establish when there is a contamination. We can anyway define a “clear” case of contamination, that is when the ratio xyl/ara is higher than one
- the presence of proteinaceous materials is unknown and there seems to be no contamination
- proteinaceous materials are simultaneously present, their source is known, and there seems to be no contamination
- the polychromy is on a wooden/paper support, contains straw, the xylose/arabinose ratio is higher than 1, or the samples are clearly contaminated by sugars originating from plant tissues present in the environmental particulate matter

**Figure 1:**
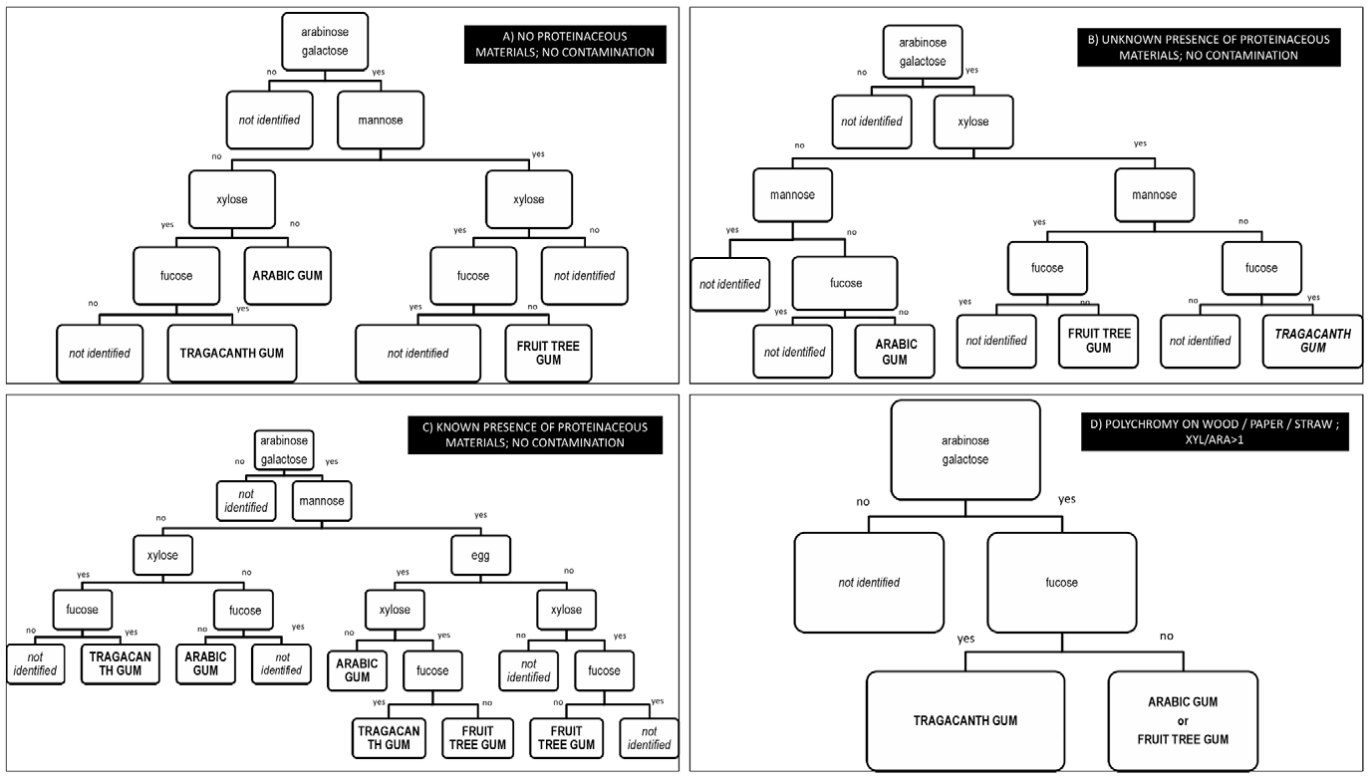
New decisional schemes to identify a plant gum in a paint sample. Legend to Figure 1. A: *there are no proteinaceous materials simultaneously present and there seems to be no contamination*; B: *the presence of proteinceous materials is unknown and there seems to be no contamination*; C: *proteinaceous materials are simultaneously present, their source is known, and there seems to be no contamination*; D: *the polychromy is on a wooden/paper support, contains straw, the xylose/arabinose ratio is higher than 1, or the samples are clearly contaminated by sugars originating from plant tissues present in the environmental particulate matter*.

The schematic representations of these decisional schemes are reported in Figure 1.

It is important to note that in the scheme only egg is taken into consideration among the proteinaceous media, as it is the only one that causes qualitative modification of the sugar profiles of the three plant gums, as discussed previously. Moreover, only fruit tree, arabic, and tragacanth gums are considered in this decisional scheme as they are the most commonly used; taking into consideration all possible materials (gums, contaminants and other organic binders present in an art object) would lead to an infinite number of possibilities that cannot be included in any form of scheme. Of course, as it should always be done in the case of analytical chemistry, data must be carefully evaluated and the results of the application of a model to help interpreting the data (such as a decisional scheme, or a statistical treatment of the data) must be critically weighed.

### 4. The analysis of paint samples – data interpretation

Hundreds of paint samples have been analyzed for plant gums at the two laboratories over the years, and a sub-set of 53 samples was chosen as representative case studies and is discussed here. The sugar profiles are reported in Table 7. Before the extension of the database [28], and the study of the effects of the simultaneous presence of inorganic and proteinaceous media, a positive identification of the saccharide materials was not possible for most of the samples; only 7% of the samples containing polysaccharide binders could be identified by comparing the qualitative and quantitative sugar profiles to reference gums and 93% of the samples showed sugar profiles that did not match those of the reference gums, either from a quantitative or qualitative point of view. In fact, several plant gums had correlation coefficients higher than 0.9 to the same art object. Similarly, the source of the saccharide material remained unidentified by using the decisional scheme. For a more detailed discussion on this, see Supporting Information S1.

**Table 7:** Data interpretation of the sugar composition of the samples analyses in the case a): there are no proteinaceous materials simultaneously present and there seems to be no contamination.

origin	Artwork	sample	analytical procedure	sugar content (DCCI: µg) (GCI %)	xylose	arabinose	rhamnose	fucose	galacturonic acid	glucuronic acid	mannose	galactose	glucose	proteinaceous material identified*	correlation coefficient	decisional scheme: - result	conclusions
Near Huaca de La Luna, Peru	mural painting, El Brujo	P-b m	GCI	0.47	1.1	43.8	5.6	1.4	-	-	3.1	44.9	yes	no	0.95 A; 0.95 F	N/I	not identified
		P t m	GCI	0.003	11.1	33.3	29.6	11.1	-	-	3.7	11.1	no	no	0.89 A	N/I	not identified
The Metropolitan Museum of Art, New York, USA	painting “Beggar No. 1”, by Jacob Lawrence, tempera on paper	1938 JL u	GCI	4.11	0.5	35.5	17.0	0.0	-	-	0.5	46.5	yes	no	0.99 A	A	arabic gum
National Museum of American Art, Washington, D. C., USA	painting “New Jersey”, by Jacob Lawrence, gouache on paper	1946 JL r	GCI	13.74	0.5	32.8	12.9	0.1	-	-	0.2	53.6	yes	no	0.99 A	A	arabic gum
Detroit Institute of Arts, Detroit, MI, USA	painting “Composition for Clarinets and Tin Horn”, by Ben Shahn, tempera on panel	1951 BS	GCI	3.07	0.7	37.4	13.9	0.1	-	-	0.6	47.3	yes	no	0.99 A	A	arabic gum
Temple of the Winged Lions, Petra, Jordan	polychromy on pottery	P-b	GCI	0.06	23.1	36.7	21.9	5.5	-	-	3.9	9.0	yes	no	0.95T	N/I	not identified
Tomb 826, Petra, Jordan	mural painting	P-CO82654B	GCI	0.08	6.1	12.7	7.4	2.0	-	-	21.7	50.0	yes	no	no	N/I	not identified

*F fruit tree gum; T tragacanth gum; A arabic gum; E egg; M milk; G animal glue; N/A = not analysed.; N/I = not identified; proteinaceous media were analysed at DCCI according to Lluveras et all* [*35*] *, and at GCI according to Shilling et all* [*36*] *.*

**Figure 2:**
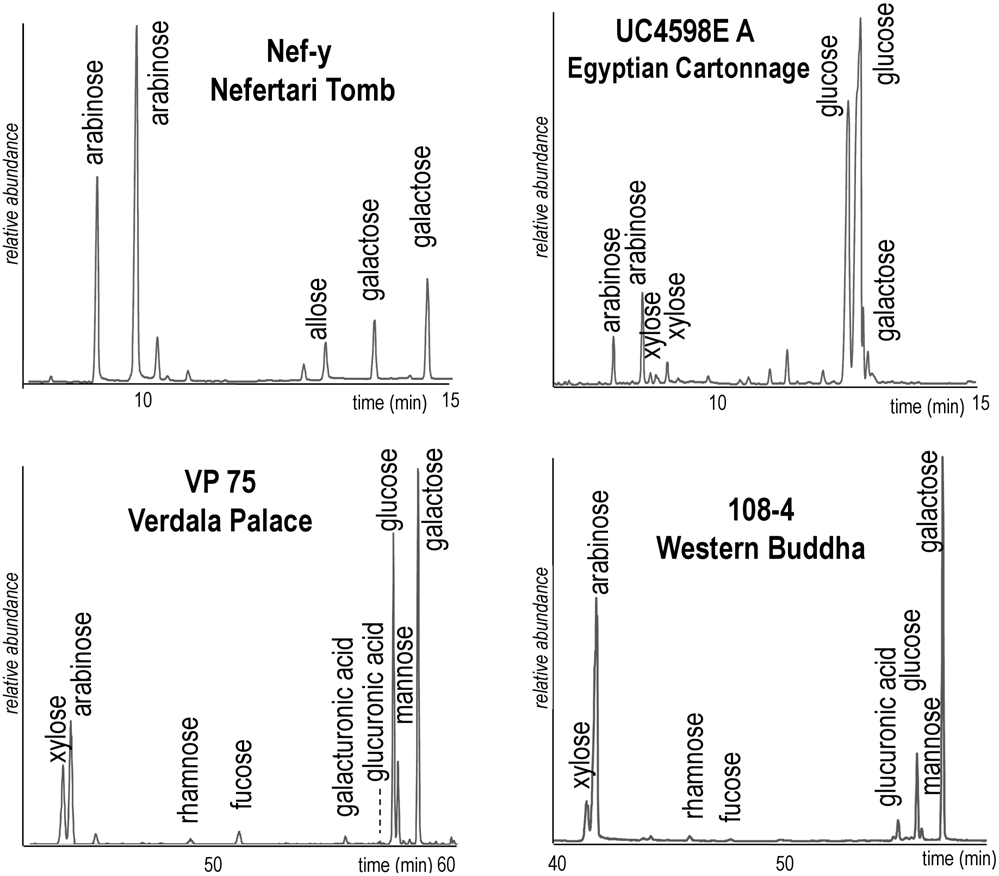
GC-MS chromatograms of 4 paint samples. Legend to Figure 2. Samples Nef-y and UC4598EA were analysed according to the GCI procedure and samples VP75 and 108-4 according to the DCCI procedure.

In the light of the new database and decisional schemes built over the course of this research, the sugar composition of the paint samples can be reinterpreted and show a much higher level of success. Results are summarised in Table 7, Table 8, Table 9, and Table 10, and are divided according to the cases listed in Paragraph 3.3 and the decisional scheme used. The correlation coefficients were calculated with respect to all reference arabic, tragacanth, and fruit tree unpigmented and pigmented paint replicas, including the artificially aged samples. The last column of the table reports the final identification of the source of the saccharide materials present in each sample, based on the results of the different identification methods, and when possible the whole organic composition of the sample is taken into account using critical evaluation of the data and possible contamination sources. A detailed discussion of each work of art is reported at the end of the table. The chromatograms of four of the painting samples are shown in Figure 2.

**Table 8:** Data interpretation of the sugar composition of the samples analysed in the case b): the presence of proteinaceous materials is unknown and there seems to be no contamination.

origin	Artwork	sample	analytical procedure	sugar content (DCCI: µg) (GCI %)	xylose	arabinose	rhamnose	fucose	galacturonic acid	glucuronic acid	mannose	galactose	glucose	proteinaceous material identified*	correlation coefficient	decisional scheme: - result	conclusions
Near Huaca de La Luna, Peru	mural painting, El Brujo	P-r m	GCI	0.61	0.6	58.4	6.2	1.2	-	-	0.6	32.9	yes	yes, N/I	0.97 F	N/I	not identified
Constantinople, Turkey	Manuscript	C u	GCI	6.01	0.0	74.1	0.8	0.0	-	-	0.0	25.0	yes	NA	0.99 F, 0.99 A	A	arabic gum
Cave 85, Mogao China	mural painting	MS-4-D	GCI	7.50	3.8	29.5	41.1	1.0	-	-	8.1	16.6	yes	NA	no	N/I	not identified
		M15 -5	GCI	4.07	13.7	19.1	14.5	8.8	-	-	8.9	35.0	yes	NA	0.94 F	N/I	not identified
		MS-2-U	GCI	0.07	11.9	15.5	17.2	9.4	-	-	8.8	37.2	yes	NA	0.87 A	N/I	not identified
		M-o.red	GCI	0.77	17.1	35.8	2.7	5.2	-	-	4.4	34.8	yes	NA	0.98 F	N/I	not identified
Nefertari Tomb, Luxor, Egypt	mural painting	Nef-y	GCI	2.04	0.6	69.0	1.0	0.0	-	-	0.0	29.4	no	NA	0.99 A, 0.98 F	A	arabic gum
		Nef-r	DCCI	18.4	0.0	61.1	0.0	0.0	0.0	0.0	0.0	38.9	no	NA	0.99 A, 0.99 F	A	arabic gum
		Nef-b	DCCI	0.4	0.0	49.2	1.5	0.0	0.0	0.0	0.0	49.4	no	NA	0.99 A, 0.97 F	A	arabic gum
Vardala Palace, Malta	painted architecture	VP75	DCCI	9.5	8.2	61.6	2.6	0.7	0.7	5.9	0.8	19.5	yes	NA	0.99 F	N/I	fruit tree gum

*F fruit tree gum; T tragacanth gum; A arabic gum; E egg; M milk; G animal glue; N/A = not analysed.; N/I = not identified; proteinaceous media were analysed at DCCI according to Lluveras et all* [*35*] *, and at GCI according to Shilling et all* [*36*] *.*

**Table 9:** Data interpretation of the sugar composition of the samples analysed in the case c): proteinaceous materials are simultaneously present, their source is known, and there seems to be no contamination.

origin	Artwork	sample	analytical procedure	sugar content (DCCI: µg) (GCI %)	xylose	arabinose	rhamnose	fucose	galacturonic acid	glucuronic acid	mannose	galactose	glucose	proteinaceous material identified*	correlation coefficient	decisional scheme: - result	conclusions
Chiesa della Missione, Mondovi, Italy	mural painting by A. Pozzo	M-op	GCI	1.40	2.4	18.3	9.9	1.3	-	-	29.9	38.2	yes	E	no	T	tragacanth gum
Byzantine Christian Museum of Greece, Athens, Greece	panel painting “Panagia Kardiotissa”, Aggelos	pkB2	DCCI	0.5	0.0	8.6	0.0	0.0	0.0	0.0	54.8	36.6	yes	E	no	A	arabic gum
Western Buddha, Bamiyan Valley, Afghanistan	polychromy on clay sculpture, fragment	168-2	DCCI	0.2	58.9	0.0	0.0	0.0	0.0	0.0	0.0	41.1	yes	E	no	N/I	not identified
		108-4	DCCI	8.0	11.1	18.1	2.1	2.7	1.5	4.4	14.2	45.9	yes	E	0.92 F	T	tragacanth gum
		108-3	DCCI	1.8	17.2	18.8	0.0	2.6	0.8	1.3	10.4	48.9	yes	E	0.91 F	T	tragacanth gum
Buddhist temple of Shuilu'an (Shaanxi Province, China)	polychromy on clay sculpture	SL-R10 g	DCCI	0.3	51.6	0.0	0.0	0.0	0.0	0.0	36.3	12.1	yes	E+G	no	N/I	no polysaccharide binder
		SL-R10 r	DCCI	1.1	15.6	49.6	0.0	0.0	0.0	0.0	19.3	15.5	yes	E+G	0.90 F	F	fruit tree gum
		SLGR02	DCCI	0.3	0.0	0.0	10.7	5.0	0.0	5.1	45.7	33.5	yes	M	no	N/I	no polysaccharide binder

*F fruit tree gum; T tragacanth gum; A arabic gum; E egg; M milk; G animal glue; N/A = not analysed.; N/I = not identified; proteinaceous media were analysed at DCCI according to Lluveras et all* [*35*] *, and at GCI according to Shilling et all* [*36*] *.*

**Table 10:** Data interpretation of the sugar composition of the samples analysed in the case d): the polychromy is on a wooden/paper support, contains straw, the xylose/arabinose ratio is higher than 1, or the samples are clearly contaminated by sugars originating from plant tissues present in the environmental particulate matter.

origin	Artwork	sample	analytical procedure	sugar content (DCCI: µg) (GCI %)	xylose	arabinose	rhamnose	fucose	galacturonic acid	glucuronic acid	mannose	galactose	glucose	proteinaceous material identified*	correlation coefficient	decisional scheme: - result	conclusions
Huaca de La Luna, Peru	polychromy on wood	P-v	GCI	1.05	17.5	12.5	14.2	0.3	-	-	9.5	46.1	yes	NA	0.84 F	F or A	
		P-o	GCI	0.15	70.6	4.7	3.0	0.0	-	-	11.0	10.7	yes	NA	No	F or A	uncertain
		P-o	DCCI	14.9	32.9	4.9	4.8	0.0	0.0	0.0	19.6	37.8	yes	NA	No	F or A	uncertain
		P-c	GCI	0.15	24.5	25.1	4.3	0.0	-	-	9.4	36.8	yes	NA	0.9 F	F or A	uncertain
		P-c	DCCI	5.8	16.8	15.2	5.4	0.7	0.0	0.0	15.4	46.4	yes	NA	0.85 F	F or A	uncertain
Cave 85, Mogao China	mural painting	MS-8-D	GCI	0.04	69.8	5.2	0.1	0.2	-	-	20.9	3.9	yes	NA	no	F or A	not identified
Egypt	Cartonnage	UC4598E-A	GCI	1.18	13.6	56.4	1.1	0.1	-	-	1.4	27.4	yes	NA	0.99 A, 0.99 F	F or A	arabic gum
Egypt	Cartonnage	USC9429-g	GCI	14.14	0.0	87.7	0.0	0.0	-	-	0.0	12.3	no	yes, N/I	0.98 A, 0.98 F, 0.98 T	A	arabic gum
		USC9429-c	GCI	17.13	0.0	82.9	0.3	0.0	-	-	0.1	16.7	no	NA	0.98 A, 0.98 F, 0.98T	A	arabic gum
		USC9429-y	GCI	2.25	4.8	60.5	0.0	0.0	-	-	2.0	32.7	yes	NA	0.99 A, 0.99 F	F or A	arabic gum
Egypt	polychromy on wood ushabti	USC9402	GCI	17.04	84.5	1.1	4.3	0.4	-	-	4.0	5.8	yes	no	no	F or A	fruit tree gum or arabic gum
Egypt	polychromy on wooden male head (Skirball	A939	GCI	7.50	35.2	7.8	16.7	6.0	-	-	13.4	20.9	yes	G	no	N/I	not identified
Tomb of Saint Anthony, Basilica of Saint Anthony from Padua, Padua, Italy	dark painted decoration on marble high-relief “St. Anthony receiving the Franciscan habit”, by Antonio Minello	Pdv 3	DCCI	1.2	29.6	13.7	2.6	0.9	0.0	15.1	22.8	15.3	yes	E	no	F or A	fruit tree gum or arabic gum
	dark painted decoration on marble high-relief “The young man resurrected by the Saint”, by Danese Cattaneo, completed by Girolamo Campagna	Pdv 4	DCCI	0.6	46.2	26.2	0.0	0.0	0.0	0.0	10.6	17.1	yes	E	0.86 T	F or A	fruit tree gum or arabic gum
	dark painted decoration on marble high-relief “The miracle of the reattached foot”, by Tullio Lombardo	Pdv 6	DCCI	1.3	83.8	8.1	0.0	0.0	0.0	0.0	3.7	4.4	yes	E	no	F or A	fruit tree gum or arabic gum
Monumental Cemetery, Pisa, Italy	mural painting, superficial organic patina	patina superficial	DCCI	2.7	27.8	19.8	5.0	0.4	0.0	2.9	15.9	28.3	yes	NA	no	F or A	fruit tree gum or arabic gum
Byzantine Christian Museum of Greece, Athens, Greece	panel painting “Panagia Kardiotissa”, Aggelos	pk2	DCCI	0.5	42.3	7.8	2.4	3.4	0.0	0.0	0.0	17.8	yes	M	no	T	tragacanth gum
		pk4	DCCI	1.7	11.0	4.7	2.6	2.5	1.3	1.8	30.1	46.0	yes	E+G+M	no	T	tragacanth gum
		pk8	DCCI	1.2	51.3	6.3	2.5	0.0	0.0	0.0	16.2	23.7	yes	G	no	F	fruit tree gum
		pk9	DCCI	13.3	27.7	2.6	5.4	3.1	0.0	0.0	43.9	17.3	yes	E	no	T	tragacanth gum
Eastern Buddha, Bamiyan Valley, Afghanistan	restoration patina on polychrome clay sculpture	214-int	DCCI	2.8	20.4	6.2	2.4	1.9	0.0	1.1	24.6	43.4	yes	E	no	T	tragacanth gum
	polychromy on clay sculpture	214-6-5	DCCI	5.1	11.2	7.4	2.8	2.2	1.0	1.9	23.5	49.9	yes	E	no	T	tragacanth gum
		214-4-3	DCCI	0.7	42.3	10.8	0.0	0.0	0.0	0.0	13.3	33.6	yes	E	no	F or A	fruit tree gum or arabic gum
Western Buddha, Bamiyan Valley, Afghanistan	polychromy on clay sculpture, fragment	97-2	DCCI	0.3	67.6	13.9	0.0	0.0	0.0	0.0	0.0	18.5	yes	M	no	N/I	not identified
		14-7-5-4	DCCI	1.3	36.2	7.8	3.4	3.9	0.0	2.4	31.9	14.4	yes	E	no	T	tragacanth gum
Buddhist temple of Shuilu'an (Shaanxi Province, China)	polychromy on clay sculpture	SL-R10 w	DCCI	2.6	14.9	12.5	22.2	8.1	0.0	4.6	21.8	16.0	yes	E+G	no	T	tragacanth gum
		SL-R10 c	DCCI	0.7	13.8	9.2	16.3	4.0	0.0	0.0	35.9	20.9	yes	E+G	no	T	
		R08B	DCCI	1.2	46.4	7.0	0.0	0.0	0.0	0.0	20.1	26.4	yes	M+G	no	F or A	fruit tree or arabic gum

*F fruit tree gum; T tragacanth gum; A arabic gum; E egg; M milk; G animal glue; N/A = not analysed.; N/I = not identified; proteinaceous media were analysed at DCCI according to Lluveras et all* [*35*] *, and at GCI according to Shilling et all* [*36*] *.*

#### Polychromy on wood (10^th^ century) and Peru (decisional schemes B and D)

Results obtained on these South American paintings point to a fruit tree or arabic gums. The fact that fruit trees and Acacia plants are not native to the area invalidate the identification. We present these samples as an example in which the use of the decisional scheme does not make sense, because the work of art comes from an area that was geographically isolated. The gums on this polychromy on wood could be due to local plant gums such as mesquite tree or cashew gum.

#### Mural painting (El Brujo; 100 BC-650 AD) (decisional schemes B)

The presence of all sugars and the absence of data about the proteinaceous materials do not allow the identification of the gum source.

#### Manuscript (13^th^ century) Constantinople, Turkey

The sugar profile is in perfect agreement with that of an arabic gum obtained from the sap of, for example; *Acacia giraffe*, *Acacia karoo*, or *Acacia seyal* trees [28]. As this manuscript is on paper, glucose is most likely a contamination from the support.

#### Tempera on paper (“Beggar No. 1”, by Jacob Lawrence, 1938), gouache on paper (“New Jersey”, by Jacob Lawrence, 1946), and tempera on panel (“Composition for Clarinets and Tin Horn”, by Ben Shahn, 1951)

The sugar profiles are perfectly in agreement with that of an arabic gum obtained from the sap of *Acacia senegal* [28]. Glucose is most likely a contamination from the support.

#### Mural painting, Cave 85, 6^th^ century, Mogao, China

The sugar composition of these samples cannot be identified. Most samples contain all the sugars and the quantitative profiles do not match any of the reference samples. The reasons for this is that the earthen preparation layer contains degraded straw and a large growth of an ubiquitous white fluffy fungus (containing high relative amounts of glucose, mannose, and galactose [28]). Moreover, one of the samples (M.o.red) contained an organic colorant, which is a source of further contamination of the sugar profile.

#### Mural painting, 13^th^ century BC, Nefertari Tomb, Luxor, Egypt

The sugar profiles obtained with both analytical procedures, are perfectly in agreement with that of an arabic gum obtained from the sap of, for example; *Acacia giraffe, Acacia karoo*, or *Acacia seyal* trees [28]

#### Cartonnage (about 100 AD), cartonnage (512-351 BC), Egypt

As mannose (fruit tree marker) and fucose (tragacanth gum marker) are missing in some of the samples, and the paintings are on a cartonnage, it is possible to suppose that xylose, comes from the degraded support, and the saccharide binder is an arabic gum obtained from the sap of, for example; *Acacia giraffe*, *Acacia karoo*, or *Acacia seyal* trees [28]

#### Polychromy on wooden male head (Skirball, about 100 AD), Polychromy on wood ushabti, Egypt

The samples were collected from wooden supports, so it cannot be excluded that sugars from its degradation are contaminating the sample. There were no other samples analyzed from the same object, so it is impossible to identify the source of the saccharide binder without the knowledge of other possible organic materials that may be present.

#### Polychromy on pottery (Temple of the Winged Lions, 1st century), mural painting (Tomb 826, Petra, 1^st^ century) Petra, Jordan

The presence of all sugars without the knowledge of any other possible organic material present makes it impossible to identify the source of the saccharide binder.

#### Mural painting by A. Pozzo (17^th^ century) Chiesa della Missione, Mondovi, Italy

This sample contains egg and cochineal as red dye, which both contribute to the sugar profile with mannose [28]. On this basis the presence of tragacanth gum can be reasonably hypothesised.

#### Painted architecture (1910) Vardala Palace, Malta

This sample does not present a clear profile, although the presence of relatively high amounts of glucuronic acid and the correlation coefficient seem to suggest the presence of a fruit tree gum.

#### Painted decorations on marble high-relief (“St. Anthony receiving the Franciscan habit”, by Antonio Minello, 1517; “The young man resurrected by the Saint”, by Danese Cattaneo and Girolamo Campagna, 1573; The miracle of the reattached foot”, by Tullio Lombardo, 1504) Tomb of Saint Anthony, Basilica of Saint Anthony from Padua, Padua, Italy

Considering the relatively high level of xylose together with the presence of glucose, that clearly point to a contamination, the sugar profile of these samples are in agreement with both arabic and fruit tree gum.

#### Superficial organic patina on a mural painting (20^th^ century) Monumental Cemetery, Pisa, Italy

As in the previous case, the qualitative sugar profile points to the presence of a fruit tree or arabic gum.

#### Panel painting (“Panagia Kardiotissa”, by Aggelos 15^th^ century) Byzantine Christian Museum of Greece, Athens, Greece

Tragacanth, fruit tree, arabic gum, and egg are the binders of the different layers. The samples presented here in fact contain different paint layers, some original, others belonging to at least to two different restorations

#### Polychrome clay sculptures (Eastern and Western Buddhas, 6^th^ century), Bamiyan Valley, Afghanistan

Although samples 214-4-3, 97-2, and 168-2 do not present a clear sugar composition, tragacanth gum was identified in the restoration and original paint layers. This identification is supported by the fact that tragacanth gum is native to the Middle East.

#### Polychrome clay sculpture (Buddhist temple of Shuilu'an, 16^th^ century) Shaanxi Province, China

Sample SL-R10 g shows the simultaneous presence of egg and animal glue, the absence of arabinose, and the high relative content of xylose (probably coming from contamination). This suggests that in this layer there is no polysaccharide binder and that the sugars present are due to the proteinaceous materials and contaminants; in sample SLGR02 the presence of milk, the absence of arabinose, and the high relative content of xylose all suggest that in this layer there is no polysaccharide binder and that the sugars present are due to proteinaceous materials and contaminants; sample SL-R10 c contains fruit tree gum; samples SL-R10 w and SL-R10 c contain egg contributing to the sugar profile with mannose, thus tragacanth gum can be reasonably hypothesised; and finally in sample R08B the presence of arabic or fruit tree gum can be hypothesised.

## Conclusions

In this paper, the effect of the simultaneous presence of proteinaceous binders, plant tissues, and inorganic materials on the sugar profile of arabic, tragacanth, and fruit tree gums was systematically investigated. The study revealed for the first time in the literature that protein, plant tissues, and inorganic materials are responsible for the modified sugar profiles, both quantitatively and qualitatively, and this must be taken into consideration when evaluating the data obtained from a paint sample. In particular it was possible to highlight that:

arabinose, fucose and galactose are relatively stable upon ageing, although subject to some changes in the relative amounts depending on the pigment and ageing parameters,
xylose is relatively stable upon ageing, although subject to changes in the relative amounts depending on the pigment and ageing parameters. Moreover it is present and abundant in plant tissues that can contaminate a painted object. When the ratio xyl/ara is higher than one and glucose is present, then there is definitely contamination and xylose can no longer be used to distinguish arabic from fruit tree and tragacanth gums
mannose is relatively stable upon ageing, although subject to changes in the relative amounts depending on the pigment and ageing parameters. Despite this, it cannot be considered the marker of fruit tree gum as it can be also due to the simultaneous presence of egg or to contamination from some plant tissues
rhamnose seems more subject to changes in its relative content, as fruit tree and tragacanth gums relative content can be below 1%;
galacturonic and glucuronic acids appear quite unstable with ageing, and they cannot be used to distinguish between one gum and another

On this basis, a new model was developed in order to help interpret the sugar profile of a paint sample, and resulted in four different decisional schemes based on the presence/absence of arabinose, galactose, mannose, xylose and fucose using the following considerations:

- there are no proteinaceous materials simultaneously present and there seems to be no contamination
- the presence of proteinaceous materials is unknown and there seems to be no contamination
- proteinaceous materials are simultaneously present, their source is known, and there seems to be no contamination
- the polychromy is on a wooden/paper support, contains straw, the xylose/arabinose ratio is higher than 1, or the samples are clearly contaminated by sugars originating from plant tissues present in the environmental particulate matter

This new model was used to interpret the data obtained from the analysis of the samples from works of art, allowing a positive interpretation of the source of the saccharide material found in 52% of the cases (compared to the 7% obtained based on previous knowledge). Moreover, in 22% of the cases, a suggestion of the source of the saccharide material is also given. This resulted in only 24% of the samples where the saccharide material was not identified, often due to incomplete knowledge on the composition of other organic materials simultaneously present.

## Supporting Information

Supporting Information S1 **The characterisation of saccharide materials in the paint samples, according to the knowledge previous to this research.**
(DOCX)
