# Supplementary material for: The Development of a New Analytical Model for the Identification of Saccharide Binders in Paint Samples
Source: PLoS One. 2012 Nov 14;7(11):e49383. doi: 10.1371/journal.pone.0049383 (PMC3498129; doi:10.1371/journal.pone.0049383)
Supplement: Supporting Information S1 — The characterisation of saccharide materials in the paint samples, according to the knowledge previous to this research. (DOCX) [file pone.0049383.s001.docx]

**Supporting information**

**The characterisation of saccharide materials in the paint samples, according to the knowledge previous to this research**

To distinguish between one polysaccharide gum and another, there are two conventional methods that can be used. The first one is to use a decisional scheme which takes into consideration all sugars in the chromatogram but glucose as it may derive from several sources, i.e. not just plant gums (tragancanth is the only gum that contains small amounts of glucose) [1]. Another way of interpreting the data is to evaluate the correlation between the monosaccharide composition of the sample with the database available. This method provides a correlation coefficient which indicates the quality of the correlation. A correlation coefficient of 1.00 or 0.99 indicates an excellent correlation; values between 0.96 and 0.98 indicate a good correlation, and values lower than 0.96 indicate a poor correlation.

The sugar profiles of the paint samples analysed and the gum identifications obtained using the decisional scheme previously reported in the literature [1] and the correlation coefficient calculated comparing the sugar profiles of the paint samples to the those of unpigmented raw arabic, tragacanth and fruit tree gums, are shown in Table 1

Table 1. Data interpretation of the sugar composition of the samples analysed using the correlation coefficients and the decisional scheme previously reported in the literature.

| **sample** | **analytical procedure** | **sugar content**  **(DCCI: µg)**  **(GCI %)** | **sugar composition** | | | | | | | | | **identification**  **according to previous knowledge**  **(paint samples I)** | |
| --- | --- | --- | --- | --- | --- | --- | --- | --- | --- | --- | --- | --- | --- |
|  |  |  |  |  |  |  |  |  |  |  |  |  |  |
|  |  |  | **xylose** | **arabinose** | **rhamnose** | **fucose** | **galacturonic acid** | **glucuronic acid** | **mannose** | **galactose** | **glucose** | **correlation**  **coefficient** | **decisional**  **scheme** |
| P-v | GCI | 1.05 | 17.5 | 12.5 | 14.2 | 0.3 | - | - | 9.5 | 46.1 | yes | no | F |
| P-o | GCI | 0.15 | 70.6 | 4.7 | 3.0 | 0.0 | - | - | 11.0 | 10.7 | yes | no | F |
| P-o | DCCI | 14.9 | 32.9 | 4.9 | 4.8 | 0.0 | 0.0 | 0.0 | 19.6 | 37.8 | yes | no | no |
| P-c | GCI | 0.15 | 24.5 | 25.1 | 4.3 | 0.0 | - | - | 9.4 | 36.8 | yes | no | F |
| P-c | DCCI | 5.8 | 16.8 | 15.2 | 5.4 | 0.7 | 0.0 | 0.0 | 15.4 | 46.4 | yes | no | no |
| P-b m | GCI | 0.47 | 1.1 | 43.8 | 5.6 | 1.4 | - | - | 3.1 | 44.9 | yes | 0.99 A; 0.98 F | no |
| P-r m | GCI | 0.61 | 0.6 | 58.4 | 6.2 | 1.2 | - | - | 0.6 | 32.9 | yes | 0.99 Ghatti | A |
| P t m | GCI | 0.003 | 11.1 | 33.3 | 29.6 | 11.1 | - | - | 3.7 | 11.1 | no | no | no |
| C u | GCI | 6.01 | 0.0 | 74.1 | 0.8 | 0.0 | - | - | 0.0 | 25.0 | yes | 0.99 A, 0.99 F | A |
| 1938 JL u | GCI | 4.11 | 0.5 | 35.5 | 17.0 | 0.0 | - | - | 0.5 | 46.5 | yes | 1 .00 A | A |
| 1946 JL r | GCI | 13.74 | 0.5 | 32.8 | 12.9 | 0.1 | - | - | 0.2 | 53.6 | yes | 0.99 A | A |
| 1951 BS | GCI | 3.07 | 0.7 | 37.4 | 13.9 | 0.1 | - | - | 0.6 | 47.3 | yes | 1 .00 A | A |
| MS-8-D | GCI | 0.04 | 69.8 | 5.2 | 0.1 | 0.2 | - | - | 20.9 | 3.9 | yes | 0.97 T | no |
| MS-4-D | GCI | 7.50 | 3.8 | 29.5 | 41.1 | 1.0 | - | - | 8.1 | 16.6 | yes | no | no |
| M15 -5 | GCI | 4.07 | 13.7 | 19.1 | 14.5 | 8.8 | - | - | 8.9 | 35.0 | yes | 0.90 A | no |
| MS-2-U | GCI | 0.07 | 11.9 | 15.5 | 17.2 | 9.4 | - | - | 8.8 | 37.2 | yes | no | no |
| M-o.red | GCI | 0.77 | 17.1 | 35.8 | 2.7 | 5.2 | - | - | 4.4 | 34.8 | yes | 0.97 F | no |
| Nef-y | GCI | 2.04 | 0.6 | 69.0 | 1.0 | 0.0 | - | - | 0.0 | 29.4 | no | 0.99 A, 0.99 F | no |
| Nef-r | DCCI | 18.4 | 0.0 | 61.1 | 0.0 | 0.0 | 0.0 | 0.0 | 0.0 | 38.9 | no | 0.99 A, 0.99 F | A |
| Nef-b | DCCI | 0.4 | 0.0 | 49.2 | 1.5 | 0.0 | 0.0 | 0.0 | 0.0 | 49.4 | no | 0.97 F | A |
| UC4598E-A | GCI | 1.18 | 13.6 | 56.4 | 1.1 | 0.1 | - | - | 1.4 | 27.4 | yes | 1.00 F | no |
| USC9429-g | GCI | 14.14 | 0.0 | 87.7 | 0.0 | 0.0 | - | - | 0.0 | 12.3 | no | 0.98 A, | no |
| USC9429-c | GCI | 17.13 | 0.0 | 82.9 | 0.3 | 0.0 | - | - | 0.1 | 16.7 | no | 0.99 A | no |
| USC9429-y | GCI | 2.25 | 4.8 | 60.5 | 0.0 | 0.0 | - | - | 2.0 | 32.7 | yes | 0.99 F | no |
| USC9402 | GCI | 17.04 | 84.5 | 1.1 | 4.3 | 0.4 | - | - | 4.0 | 5.8 | yes | no | no |
| A939 | GCI | 7.50 | 35.2 | 7.8 | 16.7 | 6.0 | - | - | 13.4 | 20.9 | yes | no | no |
| P-b | GCI | 0.06 | 23.1 | 36.7 | 21.9 | 5.5 | - | - | 3.9 | 9.0 | yes | 0.80 T | no |
| P-CO82654B | GCI | 0.08 | 6.1 | 12.7 | 7.4 | 2.0 | - | - | 21.7 | 50.0 | yes | no | no |
| M-op | GCI | 1.40 | 2.4 | 18.3 | 9.9 | 1.3 | - | - | 29.9 | 38.2 | yes | no | no |
| VP75 | DCCI | 9.5 | 8.2 | 61.6 | 2.6 | 0.7 | 0.7 | 5.9 | 0.8 | 19.5 | yes | 0.93 F | no |
| Pdv 3 | DCCI | 1.2 | 29.6 | 13.7 | 2.6 | 0.9 | 0.0 | 15.1 | 22.8 | 15.3 | yes | no | F |
| Pdv 4 | DCCI | 0.6 | 46.2 | 26.2 | 0.0 | 0.0 | 0.0 | 0.0 | 10.6 | 17.1 | yes | no | no |
| Pdv 6 | DCCI | 1.3 | 83.8 | 8.1 | 0.0 | 0.0 | 0.0 | 0.0 | 3.7 | 4.4 | yes | no | no |
| patina superficial | DCCI | 2.7 | 27.8 | 19.8 | 5.0 | 0.4 | 0.0 | 2.9 | 15.9 | 28.3 | yes | no | F |
| pk2 | DCCI | 0.5 | 42.3 | 7.8 | 2.4 | 3.4 | 0.0 | 0.0 | 0.0 | 17.8 | yes | no | no |
| pk4 | DCCI | 1.7 | 11.0 | 4.7 | 2.6 | 2.5 | 1.3 | 1.8 | 30.1 | 46.0 | yes | no | no |
| pk8 | DCCI | 1.2 | 51.3 | 6.3 | 2.5 | 0.0 | 0.0 | 0.0 | 16.2 | 23.7 | yes | no | no |
| pk9 | DCCI | 13.3 | 27.7 | 2.6 | 5.4 | 3.1 | 0.0 | 0.0 | 43.9 | 17.3 | yes | no | no |
| pkB2 | DCCI | 0.5 | 0.0 | 8.6 | 0.0 | 0.0 | 0.0 | 0.0 | 54.8 | 36.6 | yes | no | no |
| 214-int | DCCI | 2.8 | 20.4 | 6.2 | 2.4 | 1.9 | 0.0 | 1.1 | 24.6 | 43.4 | yes | no | no |
| 214-6-5 | DCCI | 5.1 | 11.2 | 7.4 | 2.8 | 2.2 | 1.0 | 1.9 | 23.5 | 49.9 | yes | no | no |
| 214-4-3 | DCCI | 0.7 | 42.3 | 10.8 | 0.0 | 0.0 | 0.0 | 0.0 | 13.3 | 33.6 | yes | no | no |
| 97-2 | DCCI | 0.3 | 67.6 | 13.9 | 0.0 | 0.0 | 0.0 | 0.0 | 0.0 | 18.5 | yes | no | no |
| 168-2 | DCCI | 0.2 | 58.9 | 0.0 | 0.0 | 0.0 | 0.0 | 0.0 | 0.0 | 41.1 | yes | no | no |
| 14-7-5-4 | DCCI | 1.3 | 36.2 | 7.8 | 3.4 | 3.9 | 0.0 | 2.4 | 31.9 | 14.4 | yes | no | no |
| 108-4 | DCCI | 8.0 | 11.1 | 18.1 | 2.1 | 2.7 | 1.5 | 4.4 | 14.2 | 45.9 | yes | 0.92 F | no |
| 108-3 | DCCI | 1.8 | 17.2 | 18.8 | 0.0 | 2.6 | 0.8 | 1.3 | 10.4 | 48.9 | yes | 0.91 F | no |
| SL-R10 g | DCCI | 0.3 | 51.6 | 0.0 | 0.0 | 0.0 | 0.0 | 0.0 | 36.3 | 12.1 | yes | no | no |
| SL-R10 r | DCCI | 1.1 | 15.6 | 49.6 | 0.0 | 0.0 | 0.0 | 0.0 | 19.3 | 15.5 | yes | 0.83 F | no |
| SL-R10 w | DCCI | 2.6 | 14.9 | 12.5 | 22.2 | 8.1 | 0.0 | 4.6 | 21.8 | 16.0 | yes | no | no |
| SL-R10 c | DCCI | 0.7 | 13.8 | 9.2 | 16.3 | 4.0 | 0.0 | 0.0 | 35.9 | 20.9 | yes | no | no |
| SLGR02 | DCCI | 0.3 | 0.0 | 0.0 | 10.7 | 5.0 | 0.0 | 5.1 | 45.7 | 33.5 | yes | no | no |
| R08B | DCCI | 1.2 | 46.4 | 7.0 | 0.0 | 0.0 | 0.0 | 0.0 | 20.1 | 26.4 | yes | no | no |

Legend to the table: F fruit tree gum; T tragacanth gum; A arabic gum

Although saccharide materials are clearly present in the samples, they cannot not be identified in most cases. The results can be summarised as follows:

- 44% of the samples gave a good or excellent correlation with the reference profiles of gums;
- 22% of the samples showed a glycoside profile qualitatively, but not quantitatively, ascribable (using the decisional scheme) to that of arabic, tragacanth and fruit tree gums;
- 7% of the samples resulted to contain the same polysaccharide material using both the correlation coefficient and the decisional scheme.

Although in some cases an identification seems possible, a close look at the data reveals that in most cases the identifications are unacceptable. In fact only in samples C u; 1938 JL u, 1946 JL r, and 1951 BS, clearly contain arabic gum representing the 7% of the total number of samples. For the rest, the sugar profiles do not match those of the reference gums, from a quantitative, or qualitative, or both point of views, and several plant gums may give acceptable matching correlations to the same art object. The use of the correlation coefficient seems in general to be more successful in identifying a plant gum in a paint sample, then the decisional scheme as there are not many samples with the same qualitative sugar composition as the reference gums. Moreover the samples that show the same qualitative composition, in most cases show a very different quantitative profile with respect to the reference materials.

From the sample data the main issues can be highlighted as follows:

- in most cases glucuronic acids are absent, while at least one is always present in the reference gums, thus making identification less successful in the case of the samples analysed by the DCCI procedure.
- xylose shows a much higher relative abundance than in the sugar profiles of the reference arabic, tragacanth and fruit tree gums, as is reported in the literature [2,3]
- in almost all cases, glucose was present. Although the simultaneous presence of both a plant gum and a glucose-containing material, such as starch or honey, can be hypothesised, it is quite unlikely that this was the case with all 54 samples, since they came from easel and mural paintings from around the world and created over a time span going from the 13^th^ century BC to the 20^th^ century AD.
- in accordance with the literature, several samples show a relatively high content of galactose and a relatively low content of arabinose and rhamnose with respect to the reference arabic, tragacanth and fruit tree gums [2,3]^,^.

**References**

1. Lluveras A, Bonaduce I, Andreotti A, Colombini MP (2010) A GC/MS analytical procedure for the characterization of glycerolipids, natural waxes, terpenoid resins, proteinaceous and polysaccharide materials in the same paint micro sample avoiding interferences from inorganic media. Analytical Chemistry 81: 376-386.

2. Bonaduce I, Colombini MP, Lluveras A, Restivo V, Ribechini E (2007) GC-MS Characterisation of plant gums in samples from painted works of art. Journal of Chromatography A 1175 275-282.

3. Bleton J, Coupry C, Sansoulet J (1996) Approche d'etude des encres anciennes. Studies in Conservation 41: 95-108.
